# Evaluation of features for classification of wheezes and normal respiratory sounds

**DOI:** 10.1371/journal.pone.0213659

**Published:** 2019-03-12

**Authors:** Renard Xaviero Adhi Pramono, Syed Anas Imtiaz, Esther Rodriguez-Villegas

**Affiliations:** Department of Electrical and Electronic Engineering, Imperial College London, London, United Kingdom; University of Manitoba, CANADA

## Abstract

Chronic Respiratory Diseases (CRDs), such as Asthma and Chronic Obstructive Pulmonary Disease (COPD), are leading causes of deaths worldwide. Although both Asthma and COPD are not curable, they can be managed by close monitoring of symptoms to prevent worsening of the condition. One key symptom that needs to be monitored is the occurrence of wheezing sounds during breathing since its early identification could prevent serious exacerbations. Since wheezing can happen randomly without warning, a long-term monitoring system with automatic wheeze detection could be extremely helpful to manage these respiratory diseases. This study evaluates the discriminatory ability of different types of feature used in previous related studies, with a total size of 105 individual features, for automatic identification of wheezing sound during breathing. A linear classifier is used to determine the best features for classification by evaluating several performance metrics, including ranksum statistical test, area under the sensitivity-–specificity curve (AUC), F1 score, Matthews Correlation Coefficient (MCC), and relative computation time. Tonality index attained the highest effect size, at 87.95%, and was found to be the feature with the lowest p-value when ranksum significance test was performed. Third MFCC coefficient achieved the highest AUC and average optimum F1 score at 0.8919 and 82.67% respectively, while the highest average optimum MCC was obtained by the first coefficient of a 6^th^ order LPC. The best possible combination of two and three features for wheeze detection is also studied. The study concludes with an analysis of the different trade-offs between accuracy, reliability, and computation requirements of the different features since these will be highly useful for researchers when designing algorithms for automatic wheeze identification.

## Introduction

Chronic Respiratory Diseases (CRDs) affect over 15% of the world population. It is estimated that more than 235 million people suffer from asthma worldwide, with the disease causing in excess off 300,000 deaths per year [1]. The prevalence of COPD is even higher with more than 250 million cases reported annually, further resulting in over 3 million deaths globally [2]. COPD is also predicted to become the third leading cause of deaths worldwide by 2030, just behind ischaemic heart disease and cerebrovascular disease [3]. Furthermore, the economic impact of these respiratory diseases is also very high. As an illustration, in the UK they cost the National Health Service approximately 3 and 2 million GBP, for asthma and COPD, respectively [4].

Asthma and COPD can usually be characterized by symptoms such as wheezing, breathlessness, and coughing. The symptoms affect patients on a regular basis, and may become worse when performing various physical activities [1, 2]. They are often worse at night time and early mornings [1] causing sleep disruptions [5] and consequently reducing the quality of life of those affected. The worsening of symptoms in CRDs is known as *exacerbations*. Exacerbations can be life-threatening and can permanently alter respiratory system structures. In some countries, such as the UK, they are also one of the leading causes of emergency hospital admissions [6]. It is thus important to detect these symptoms at an early stage to improve patients’ quality of life as well as to reduce the number of deaths. Despite the high prevalence and detrimental effects of CRDs, specifically asthma and COPD, they continue to be heavily under-diagnosed, poorly managed, and often left untreated [1, 2]. Although both asthma and COPD are not curable, they can be managed to reduce the associated risks or slow down the disease progression.

The monitoring and identification of early symptoms is critical for the management of CRDs [7, 8]. One of the important symptoms that needs to be monitored is the occurrence of *wheezing* sounds during breathing. Wheezes are a type of abnormal breathing sound which usually has a high pitch lasting for more than 80ms [9]. A wheeze sound is described as a continuous whistling and sibilant sound superimposed on normal breathing. Wheezes are caused by the narrowing of airways, which then causes airflow limitations [10]. In a clinical setting, a doctor can perform conventional auscultation using a stethoscope to detect a wheezing sound. However, auscultation usually needs to be performed in a quiet environment and ideally with the patient in a still position, which restricts the duration and flexibility of monitoring. Further, since the respiratory symptoms can occur at home, in public, or even at night-time, this approach of using a stethoscope by a trained medical professional has got limited usability.

For day-to-day management and self-monitoring of CRDs, patients are often asked to maintain logs of events and to note down any details of the symptoms they think they might be experiencing. Although, this is considered to be a useful method for disease monitoring [11], it requires the patient to manually input symptoms occurrences, which can be subjective and is conditioned by patients compliance.

These issues could be alleviated by having a wearable system able to monitor respiratory sounds continuously; and intelligent algorithms extracting information from the signals to flag abnormalities. Automatic lung sound analysis, specifically for identification of wheezing sounds, has long been of significant research interest. Studies reviewed in [12] developed algorithms to discriminate between normal respiratory sounds and wheeze sounds. The different studies used several features with different classifiers to automatically label different respiratory events in the algorithms. The discriminatory features used to differentiate between wheeze and normal respiratory sound events are briefly discussed in the following.

Orjuela-Canon et al. [13] used MFCC as a feature vector, with length of 13 in an Artificial Neural Network (ANN) classifier. The data for this study was obtained from an online resource [14] which contains 4 crackle, 4 wheeze, and 5 normal breathing recordings. A first order Butterworth high-pass filter with cut-off frequency of 7.5 Hz was used to remove DC components followed by an eighth order Butterworth low-pass filter with cut-off frequency of 2.5 kHz, to bandlimit the signal. A Leave One Out Cross-Validation (LOOCV) method was used, achieving a 100% recognition rate for wheezing sounds and 80% accuracy to detect normal breathing sounds. Bahoura in [15] found that Mel-Frequency Cepstral Coefficient (MFCC) performed better for respiratory segments classification when compared to other features including Linear Predictive Coding (LPC) [16] and features based on Wavelet transform [17]. The assessment was performed by comparing the performance of the features using Gaussian Mixture Model (GMM), Multilayer Perceptron (MLP), and Vectorial Quantification (VQ). The best performance was obtained by using 24 MFCC with GMM, achieving 97.2% sensitivity and 94.2% specificity.

Oweis et al. [18] used 32 averaged power spectrum features with an ANN and reported an accuracy of 98.6% when performing abnormal and normal breathing sound classification, including wheeze events. Using a similar set of features with a multi layer perceptron and incremental supervised neural network for classification, [19] reported a 98% accuracy for abnormal sound classification. Mendes et al. [20] evaluated musical features to detect wheeze in respiratory sound segment. Result of this study was based on the average ten repetitions of 10-fold cross-validation on 24 recordings. The best performance was achieved by using Random Forest at sensitivity of 92.7% and specificity of 90.9%. Wisniewski and Zielinski in [21] introduced two features based on MPEG standard, Tonality Index (TI) and Audio Spectral Envelope (ASE) for the detection of wheezes in respiratory sound. A total of 260 respiratory recordings of 128ms each were used in this study, where 130 of them contain wheezes. A 10-fold cross-validation was used with Support Vector Machine (SVM) classifier, achieving Area Under Curve (AUC) performance of 0.951 for ASE and 0.905 for TI.

Aydore et al. [22] employed kurtosis, Renyi entropy, quartile frequency ratios, and Mean Crossing Irregularity (MCI) to differentiate between wheezing and non-wheezing episodes in breath sounds. The classifier used was Fischer Discriminant Analysis and the performance reported was 93.5% success rate in discriminating between 492 respiratory episodes. To differentiate between monophonic and polyphonic wheezes, MCI and multiple quartile frequency ratios were used in a study by Ulukaya et al. [23]. The classification methods used were k-Nearest Neighbour, SVM, and Naive Bayesian. It was found that k-Nearest Neighbour performed the best classification with this feature set, with an overall accuracy of 75.78% using a LOOCV scheme. Oletic et al. [24] used LPC error ratio as the main feature for wheeze classification. A total of 62 wheeze and 140 normal respiratory events were used in this study. Using a simple threshold classifier, 90.29% accuracy was achieved in differentiating between normal and wheezing sounds. Entropy features were used by Liu et al. [25] to discriminate between wheeze, stridor, crackle, and normal recordings. In this work, the entropy of each recording was extracted, following which, three features were computed from the extracted entropy. These features were then compared against a set of thresholds for classification of different sounds. On 45 recordings, this resulted in a detection accuracy of 70% for wheezes and 99% for normal lung sounds. Finally, Chamberlain et al. [26] used a power ratio in a specific frequency band to determine whether or not a recording, obtained using a custom stethoscope, contained wheeze sounds. The proposed power ratio feature was extracted as a ratio between maximum power in the 250-800 Hz frequency band and the mean power in the 60-900 Hz frequency band. Subsequently a SVM was used to determine if the recording contained wheezes. This resulted in a maximum recognition accuracy of 86%.

The studies reviewed above reported algorithms with high accuracy for wheeze and normal sound classification. However, the use of complex classification techniques such as neural networks as well as the use of multiple computationally-expensive features would limit their use in a long-term portable battery-powered symptom monitoring device. This is because such a system would have a restricted power budget. The use of a small battery in such devices would limit their computational power, if they were intended to operate over long periods of time.

In this paper, we review the different types of features used in previous related studies, individually, with a total size of 105 individual features, using a simple linear threshold to evaluate their ability to distinguish between wheeze and normal respiratory events. The objective of this paper is to benchmark the performance of features individually with a particular view to application within resource-constrained and portable battery-powered devices. This is important since complex classification methods with multiple features may not be suitable for many applications. Section Materials and methods first presents the description of features reviewed in this study, followed by details of the data used for analysis. In Section Results, the performance metrics that are used in this study are introduced, followed by detailed results of the classification performance for each feature. This section also discusses the optimum combination of features which results in the best performance for the detection of wheezing sounds, as well as the relative computation time requirements for different features. Finally, Section Discussion discusses the key observations in this study and provides insight into the suitability of different features for various applications and use cases.

## Materials and methods

To assess and compare the performance of different features to distinguish between wheeze and normal respiratory events, a comprehensive review of the existing works in literature was performed. The review focused mainly on studies which carried out classification between wheeze and normal respiratory events. Based on this, a number of different features were selected, with the inclusion criteria being that enough details about a feature were available for its implementation to discriminate between wheeze and normal respiratory events using a simple linear threshold. The details about the selected features, preprocessing stages, and classification, are discussed in this section.

### Features for wheeze classification

The features extracted for this study were selected from the works discussed in Section Introduction. Additionally, other time and spectral features, commonly used for audio processing were also considered for comparison. This resulted in a feature vector with a total size of 105 individual features, that are listed in Table 1.

**Table 1:** List of features extracted for evaluation.

Features Extracted	Size
Averaged PSD	32
Wavelet transform	20
MFCC	13
LPC coefficients	8
Percentile frequency ratio	4
Entropy-based	4
Power ratio	1
ASE flux	1
Tonality index	1
Mean crossing irregularity	1
Other time and spectral features	20

#### Averaged power spectrum

Averaged Power Spectrum was used in [18, 19] to create a feature vector of 32 coefficients, for classification using an ANN. The power spectrum of an event was computed and averaged to reduce the size of features into 32 different frequency bands between 0 Hz to 1950 Hz.

#### Wavelet transform

Features derived from continuous wavelet transform were used in [17] for classification of lung sounds using ANN. A decomposition of the signal using Daubechies with 4 vanishing moments was performed. The features were then extracted by taking the absolute mean, average power, and standard deviation of the coefficients in each sub-band, also the ratio of absolute mean of coefficients in adjacent sub-bands.

#### Mel frequency cepstral coefficients

MFCC are a set of coefficients which represent signal spectrum, and are defined as the Discrete Cosine transformed logarithm of a signal spectrum. MFCC was used in [13, 15] to distinguish between normal and respiratory sounds, including wheezes. Thirteen coefficients, MFCC-1 to MFCC-13, representative of a respiratory event were extracted from the whole event as feature vector, including the zeroth order. The number of filters in the filter-bank was set to 26 to obtain more detail on the mel-scale spectrum where the filter edges are a function of the sampling frequency.

#### Linear predictive coding

LPC is a time domain estimator of a signal based on linear combination of previous samples weighted with LPC coefficients [16, 24]. The coefficients and the prediction error of a 6^*th*^ order LPC were used as a feature vector in [16]. The features were obtained from 51.2 ms segments of each event with 12.8 ms overlap. In [16], the event classification label was obtained by using majority vote of the segment classification. LPC coef-1 to LPC coef-5 correspond to the second to sixth coefficients of the lpc filter, with the 6^*th*^ order error as the other feature. The prediction error energy of LPC was used in [24] for classification between wheeze and normal respiratory sounds. The feature used in [24] was the *E*^(0)^/*E*^(4)^ ratio, where *E*^(*k*)^ represents the prediction error energy of order *k*.

#### Percentile frequency ratios

Percentile frequency ratios were used as features in [22, 23] for lung sound classification. Percentile frequency *f*_*x*_ is defined as a frequency where the power of a signal reaches the *x* percentile of the total power. Four different ratios were used: *f*_25_/*f*_75_, *f*_25_/*f*_90_, *f*_50_/*f*_75_, and *f*_50_/*f*_90_.

#### Entropy-based

Entropy, as a feature, is a measure of how the signal frequency peaks are distributed in time. Three features based on this definition of entropy were used to discriminate between signal containing adventitious sounds and a normal signal [25]. The first feature *E*_*d*_ was the difference between the maximum and minimum value of the entropy of a signal across time. The ratio *E*_*r*_ between the maximum and minimum entropy across time was the second feature, while the third feature used was the mean of the entropy across time *E*_*m*_. Classification between wheeze and normal respiratory segments was performed in [22] by using an entropy-based feature, which was Renyi entropy. Renyi entropy is a generalisation of Shannon entropy defined as $H\left(X\right)=\frac{1}{1-\alpha }log\left(\sum_{i=1}^{n}{p_{i}}^{\alpha }\right)$. In the study, the time-series respiratory segments were regarded as a random variable *X*, where the constant *α* was set to two.

#### Power ratio

The study in [26], aiming to differentiate between wheeze and normal respiratory sounds, used a power ratio in specific frequency bands as a feature. The power ratio was computed by comparing the maximum peak in the 250-800 Hz range, with the mean power between 60-900 Hz. The labeling of breathing sounds was then done by comparing the ratio to a threshold.

#### Audio spectral envelope flux

ASE was used as feature vector in [21] to discriminate between wheezes and normal respiratory sounds. ASE was based on its description in MPEG-7 standard [27]. Isolated sound events were segmented into frames with 32 ms width and 8 ms shift. Features were first extracted separately for each segment, which were then averaged to represent a single event.

#### Tonality index

Similar to ASE, TI was used as feature vector in [21] to discriminate between wheezes and normal respiratory sounds. TI feature extraction was based on its description in MPEG-2 previously. Isolated sound events were segmented into frames with 32 ms width and 8 ms shift. Features were first extracted separately for each segment, which were then averaged to represent a single event.

#### Mean crossing irregularity

MCI was used in [23] and defined as the mean normalised standard deviation of the mean crossing intervals. These intervals were first calculated by subtracting the mean of an event and finding the intervals between the zero crossing indices. The intervals were then regarded as a random variable *X* with mean *E*(*X*) and standard deviation $\sqrt[{}]{var(X)}$.

#### Other time and spectral features

Musical [20] and other time and spectral features, commonly used in audio signal processing, were also extracted for comparison. These included frequency roll-off (85%, 90%, and 95%), frequency quartiles, root mean square (RMS) energy of the time series, spectral brightness, spectral irregularity, spectral kurtosis, spectral skewness, spectral crest factor, spectral centroid, spectral decrease, spectral flatness, spectral slope, spectral spread, zero crossing rate (ZCR), standard deviation (STD) and band power. The spectral features mentioned above were extracted by using a modified periodogram with Kaiser window of length 38.

### Data collection

For this study, a total of 38 different recordings were obtained from multiple repositories [14, 28–30] and books supplements [31, 32]. Based on the description of each recording provided by the source, the database collected consisted of sounds captured from the trachea, anterior, and posterior chest; using either a stethoscope or microphone. Out of the 38 total recordings, 28 recordings contained wheeze sounds. These were collected from patients with different pathologies, including asthma, bronchitis, COPD, and croup. Additionally, the normal respiratory sounds used in this study included tracheal and vesicular sounds. Age was not considered as a relevant variable in this study since not all of the recordings provided this information. However, this was not considered to be a major limitation because according to literature, the variations caused by age difference in automatic auscultation is too small to be clinically relevant [33], and in addition, the definition and description of wheezes also is not age-related [9, 34]. Each respiratory event was manually extracted and annotated from the recordings based on the description provided by the specific data source as ground truth. A total of 425 respiratory events were isolated across all recordings, of which 223 were labeled as wheezes, while the remaining 202 were labeled as normal respiratory sounds. Of these recordings, a random patient-independent partition of training and test set with a proportion of 50% each were created to evaluate the performance of the features individually.

### Data preprocessing

**Fig 1:**
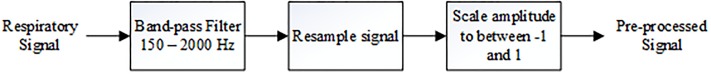
Block diagram for preprocessing steps.

Since the recordings were obtained using different equipment under also different conditions, they needed to be preprocessed to have the same fundamental characteristics in the acoustic data of respiratory sounds. As shown in Fig 1, a band-pass filter was used to filter out sounds below 150 Hz since these might have contained heart sounds, and DC offsets; and to prevent aliasing on frequencies above 2000 Hz. The band-pass filter was implemented in MATLAB as a fifth-order Butterworth filter to make the frequency response in the pass-band flat so that the filter did not affect the important respiratory information. All events collected were then resampled to 8000 Hz, as information on the respiratory sounds is bandlimited up to 2000 Hz only [35]; and processing at the lowest possible sampling frequency would reduce unnecessary computation. Amplitude scaling was performed on each event so that all samples had values between -1 and 1 without changing their mean and variance. This was performed to compensate for the intensity difference of data collected from different sources while preserving the important statistical parameters of the respiratory sounds.

### Linear classification for single feature

**Fig 2:**
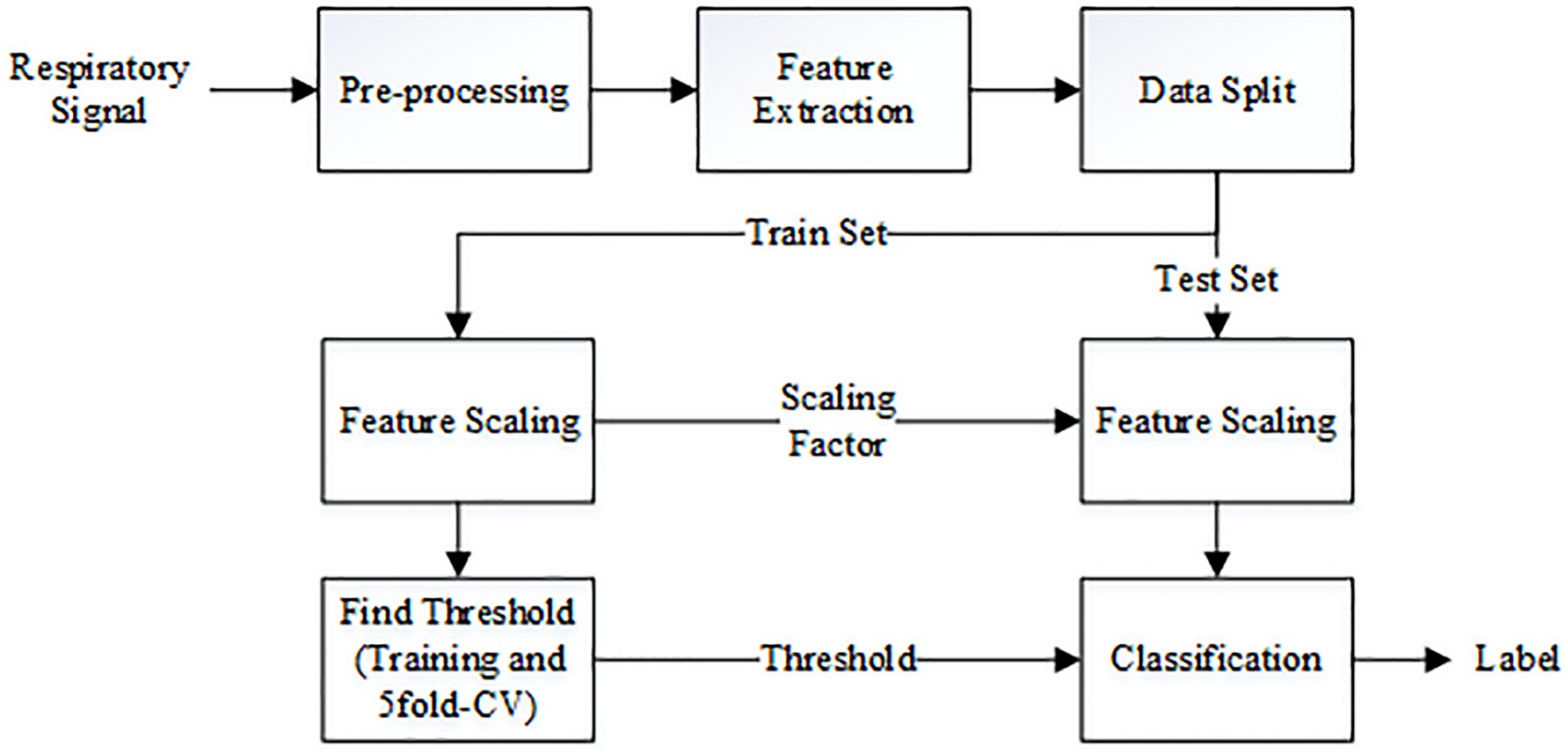
Flowchart for classification of wheeze and normal respiratory sounds using single feature.

The discriminatory ability of each feature was evaluated by using a simple linear classifier to differentiate between wheeze and normal respiratory sounds. Fig 2 shows the different stages of the evaluation method, where each respiratory event is first preprocessed before the feature extraction stage. All the features in the training set were normalized to have values between *a* = −1 and *b* = 1. Each feature *x* was scaled following Eq (1), where *x*′ is the resultant scaled feature, ranging from *a* to *b*.

$$\begin{array}{c} x^{\prime }=a+\frac{\left(b-a)(x-min(x)\right)}{max(x)-min(x)} \end{array}$$ (1)

The scaled features were then compared individually against a varied threshold to determine whether the respiratory sound contained wheezes or not.

To scale the test set, the same equation in (1) was used, but the maximum *max*(*x*) and minimum *min*(*x*) values, as scaling factors, were obtained from the training set. This was done to prevent the statistical information of the test set from leaking to the classification model. Following this, five-fold cross-validation on the training set was used to find an optimum threshold for the classifier. The performance of each feature was then measured from the test set using this optimum threshold.

### Logistic regression model for multiple features

**Fig 3:**
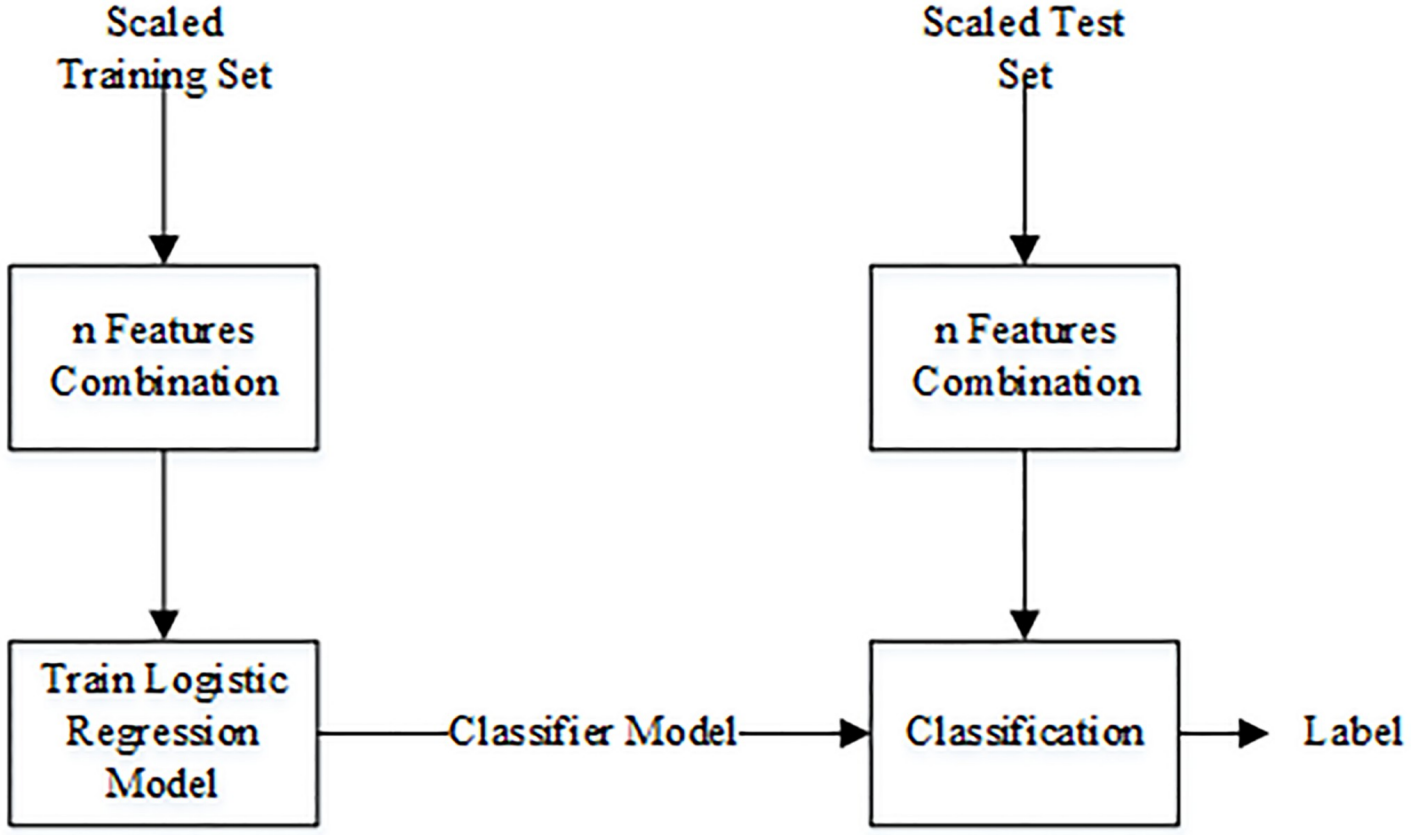
Flowchart for classification of wheeze and normal respiratory sounds using combination of features.

While the use of single features have the advantage of hugely reducing the computational load, in many applications a number of different features may have to be used to improve the classification performance. Hence, to evaluate the combination of features best suited to discriminate between wheezing and normal respiratory sounds, a binary logistic regression model was used to observe the trade off between performance and number of features used. The flowchart of the classification using a logistic regression model can be seen in Fig 3. The preprocessing, feature extraction, and feature scaling steps were the same as when a simple linear threshold was used (see previous section). The logistic regression model was trained on the scaled training set. The performance was then measured on the test set, where up to three features were used in combination. A sequential feature selection procedure was also performed using logistic regression, by iteratively adding the new feature to a feature vector to find the combination and number of features that increased the performance of the classifier.

This section presents the performance of each feature individually in discriminating between wheezing and normal breath sounds. It also shows a comparison of the features by optimizing for different objectives. The specific performance metrics used in this study are first described, followed by a detailed assessment of each feature when used in a single-feature linear classifier. This is then followed by a discussion of the best possible combination of features that can be used to improve the classification performance.

## Results

### Performance metrics

The discriminatory ability of each feature was assessed by evaluating different performance metrics from the classification results in the test data set. These metrics include Sensitivity (SE), Specificity (SP), Positive Predictive Value (PPV), and Negative Predictive Value (NPV). These are described below.

$$\begin{array}{c} SE=\frac{TP}{TP+FN} \end{array}$$ (2)

$$\begin{array}{c} SP=\frac{TN}{TN+FP} \end{array}$$ (3)

$$\begin{array}{c} PPV=\frac{TP}{TP+FP} \end{array}$$ (4)

$$\begin{array}{c} NPV=\frac{TN}{TN+FN} \end{array}$$ (5)

In these equations, TP (True Positive) represents the number of wheeze sounds correctly labeled, TN (True Negative) is the number of correctly labeled normal respiratory sounds, FP (False Positive) is the number of normal sounds mislabeled as wheezes, and FN (False Negative) is the number of wheezes which were incorrectly labeled as normal. These measures are in turn used to compute further performance metrics to compare the classification result of each feature. These metrics include the AUC of a Receiver Operating Characteristic (ROC) curve, the F1 score, and the Matthews Correlation Coefficient (MCC).

### Feature characterization using AUC

To quantify the classification performance of each feature at different detection thresholds, the ROC curves, as a function of sensitivity against specificity, were plotted, by sweeping the detection threshold of the linear classifier. This step was repeated for each feature separately, using all recordings in the database. The AUC for each feature gives an indication of which feature has a higher discriminatory ability.

**Fig 4:**
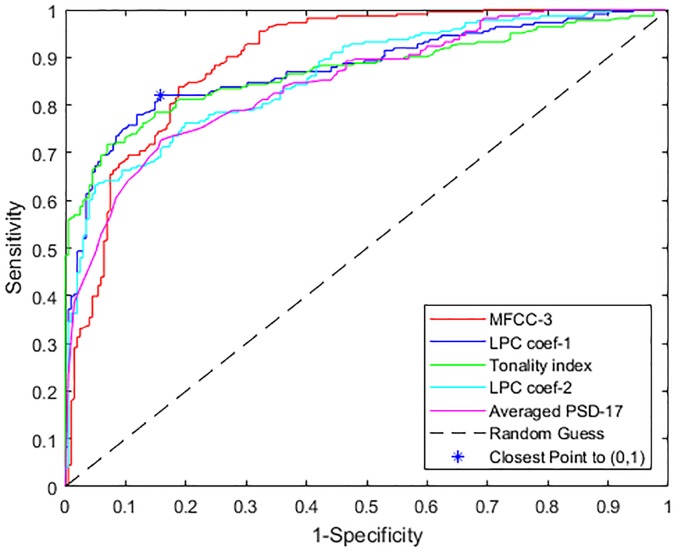
ROC of features with the highest AUC.

Fig 4 shows the ROC curves for the features with the highest values of AUC. These values are also shown in Table 2. It can be seen from these that the third MFCC coefficient resulted in the highest AUC followed by the first 6^*th*^ order LPC coefficient and tonality index. The AUC then drops for other features, showing that these three features have the highest discriminatory abilities when both sensitivity and specificity are taken into account.

**Table 2:** AUC comparison of best performing features on all dataset using linear classifier.

Feature Name	AUC
MFCC-3	0.8919
LPC coef-1	0.8708
Tonality index	0.8659
LPC coef-2	0.8557
Averaged PSD-17	0.8423
Spectral irregularity	0.8354
WT coef STD-1	0.8327
WT coef energy-1	0.8323
Averaged PSD-16	0.8300
LPC coef-3	0.8211
Spectral brightness	0.8153
95% freq roll-off	0.8147

The optimum threshold on a ROC curve is the point closest to (0,1) on the curve. For MFCC-3 which achieved the highest AUC, this point is achieved at sensitivity and specificity values of 83.86% and 81.19% respectively. The closest distance to the optimum point in the ROC curve is however achieved by the first coefficient of LPC, at 82.06% sensitivity and 84.16% specificity, shown in Fig 4 along the ROC of the feature. The sensitivity and specificity at optimum thresholds of the features with highest AUC are shown in Table 3. However, it is important to note that a ROC curve represents the trade off between sensitivity and specificity and a different point on the curve may be selected if either of these is more important in any given application.

**Table 3:** Comparison of optimum ROC points of features with the highest AUC.

Feature Name	SE	SP	Dist. to (0,1)	Threshold
MFCC-3	0.8386	0.8119	0.2479	> 0.0520
LPC coefficient-1	0.8206	0.8416	0.2393	> −0.1760
Tonality index	0.7848	0.8515	0.2615	< 0.7600
LPC coefficient-2	0.7623	0.8020	0.3094	< −0.1300
Averaged PSD-17	0.7265	0.8416	0.3161	< −0.9880
Spectral irregularity	0.7578	0.7822	0.3257	> −0.7020
WT coef STD-1	0.7578	0.7822	0.3257	< −0.7230
WT coef energy-1	0.7668	0.7673	0.3294	< −0.9510
Averaged PSD-16	0.7713	0.7723	0.3227	< −0.9800
LPC coef-3	0.6906	0.8119	0.3621	> 0.6660
Spectral brightness	0.7803	0.7673	0.3200	< −0.9120
95% freq roll-off	0.7668	0.7475	0.3437	< −0.2860

### Feature characterization using F1 score

The F1 score is a metric representing the weighted harmonic mean of sensitivity and the PPV. It is computed as shown in Eq (6). Unlike the AUC, the F1 score is used to determine the trade-off between sensitivity and PPV.

$$\begin{array}{c} F1=\frac{2(SE)(PPV)}{SE+PPV}=\frac{2(TP)}{2(TP)+FP+FN} \end{array}$$ (6)

In order to determine the optimum threshold for each feature to obtain the highest F1-score, the training dataset was used with five-fold cross-validation. This threshold was then used on the test set to compute the sensitivity and PPV, and subsequently the F1 score, individually for each feature. This process was repeated 100 times in order to further randomise the training and test dataset partition. The optimum thresholds and corresponding F1 score values on the test dataset for the highest performing features are shown in Table 4 as the average of the different repetitions. It can be seen that the top three features with highest average F1 score are MFCC-3, LPC coef-1 and, tonality index respectively. Since the F1 Score does not take specificity into account, the first one of these have significantly lower specificity values despite being ranked highly for their sensitivity and PPV.

**Table 4:** Classification result using optimum F1 score threshold on the Test-Set.

Feature Name	SE	SP	PPV	NPV	Threshold	F1 Score
MFCC-3	0.9097 ± 0.0850	0.6811 ± 0.1114	0.7628 ± 0.0478	0.8834 ± 0.0888	> −0.1409 ± 0.0969	0.8267 ± 0.0452
LPC coef-1	0.8027 ± 0.0924	0.8128 ± 0.1413	0.8359 ± 0.0881	0.7993 ± 0.0844	> −0.1990 ± 0.1163	0.8122 ± 0.0594
Tonality index	0.7657 ± 0.0915	0.8366 ± 0.0914	0.8477 ± 0.0676	0.7677 ± 0.0870	< 0.7556 ± 0.0363	0.7976 ± 0.0431
Spectral irregularity	0.8214 ± 0.0776	0.6174 ± 0.1198	0.7022 ± 0.0881	0.7644 ± 0.0894	> −0.7907 ± 0.0624	0.7495 ± 0.0381
LPC coef-2	0.8084 ± 0.1573	0.6414 ± 0.1923	0.7290 ± 0.0997	0.7846 ± 0.1236	< 0.0525 ± 0.2180	0.7491 ± 0.0729
WT coef mean-5	0.9327 ± 0.0608	0.3897 ± 0.1487	0.6291 ± 0.0609	0.8423 ± 0.1291	> −0.9154 ± 0.0339	0.7485 ± 0.0458
WT coef STD-5	0.9200 ± 0.0815	0.3910 ± 0.1593	0.6274 ± 0.0646	0.8227 ± 0.1450	> −0.8945 ± 0.0490	0.7416 ± 0.0503
WT coef energy-5	0.9145 ± 0.0956	0.4027 ± 0.1599	0.6318 ± 0.0678	0.8189 ± 0.1457	> −0.9890 ± 0.0278	0.7409 ± 0.0587
90% freq roll-off	0.8657 ± 0.1080	0.4887 ± 0.1896	0.6583 ± 0.0768	0.8008 ± 0.1553	< 0.1024 ± 0.2834	0.7401 ± 0.0492
Spectral brightness	0.8202 ± 0.1205	0.5657 ± 0.2256	0.6903 ± 0.0998	0.7715 ± 0.1269	< −0.6789 ± 0.3939	0.7369 ± 0.0533
95% freq roll-off	0.8377 ± 0.1270	0.5312 ± 0.2068	0.6745 ± 0.0863	0.7864 ± 0.1458	< −0.0306 ± 0.2682	0.7355 ± 0.0507
WT coef STD-4	0.8124 ± 0.1169	0.5415 ± 0.2130	0.6746 ± 0.0975	0.7324 ± 0.1492	> −0.6403 ± 0.1480	0.7245 ± 0.0513

### Feature characterization using MCC

MCC is a metric that is used to compare the balanced performance of the features, as it takes into account both the true and false positives and negatives. It is computed as shown in Eq (7).

$$\begin{array}{c} MCC=\frac{\left(TP)(TN)-(FP)(FN\right)}{\sqrt[{}]{\left(TP+FP)(TP+FN)(TN+FP)(TN+FN\right)}} \end{array}$$ (7)

MCC has a range between -1 and +1, where the -1 represents total disagreement between the classifier and ground truth, while +1 indicates a perfect performance. Similar to the determination of the F1 score, the MCC values were computed for each feature separately, by first determining the optimum threshold on the same training data as before, and then using this threshold with the test dataset to find the MCC value. The thresholds and average MCC values for the best performing features are shown in Table 5. The top features in this case are LPC coef-1, tonality index, and MFCC-3. Since MCC takes into account all the true and false positives and negatives, the higher ranked features achieve a relatively more balanced performance, compared to those ranked using other metrics.

**Table 5:** Classification result using optimum MCC threshold on the Test-Set.

Feature Name	SE	SP	PPV	NPV	Threshold	MCC
LPC coef-1	0.7378 ± 0.0965	0.8828 ± 0.0844	0.8825 ± 0.0672	0.7602 ± 0.0778	> −0.0918 ± 0.0902	0.6312 ± 0.0929
Tonality Index	0.6971 ± 0.0892	0.9143 ± 0.0545	0.9083 ± 0.0426	0.7358 ± 0.0780	< 0.7031 ± 0.0391	0.6269 ± 0.0727
MFCC-3	0.8630 ± 0.1223	0.7119 ± 0.1294	0.7777 ± 0.0529	0.8482 ± 0.0981	> −0.0818 ± 0.1238	0.5989 ± 0.0995
*f*_25_/*f*_90_	0.5695 ± 0.0830	0.9474 ± 0.0468	0.9292 ± 0.0572	0.6681 ± 0.0786	> 0.7388 ± 0.0633	0.5548 ± 0.0715
*f*_25_/*f*_75_	0.5141 ± 0.0773	0.9730 ± 0.0290	0.9572 ± 0.0425	0.6465 ± 0.0794	> 0.5459 ± 0.0435	0.5414 ± 0.0772
LPC coef-2	0.6664 ± 0.1345	0.8477 ± 0.1697	0.8563 ± 0.1161	0.7095 ± 0.0934	< −0.2752 ± 0.1849	0.5383 ± 0.1254
*f*_50_/*f*_90_	0.5217 ± 0.0829	0.9606 ± 0.0422	0.9397 ± 0.0532	0.6475 ± 0.0791	> 0.4959 ± 0.0664	0.5312 ± 0.0861
*f*_50_/*f*_75_	0.4807 ± 0.0773	0.9802 ± 0.0260	0.9657 ± 0.0432	0.6332 ± 0.0771	> 0.2774 ± 0.0349	0.5244 ± 0.0816
LPC coef-3	0.6284 ± 0.1366	0.8401 ± 0.1792	0.8443 ± 0.1213	0.6813 ± 0.0977	> 0.6948 ± 0.1897	0.4949 ± 0.1445
Entropy ratio	0.8863 ± 0.1488	0.3328 ± 0.3790	0.6313 ± 0.1467	0.7264 ± 0.0949	< −0.2803 ± 0.8446	0.4946 ± 0.0945
Spectral irregularity	0.6689 ± 0.1220	0.8011 ± 0.1084	0.8002 ± 0.0863	0.6949 ± 0.0844	> −0.6162 ± 0.1354	0.4816 ± 0.0605
Spectral brightness	0.6755 ± 0.1720	0.7539 ± 0.1748	0.7809 ± 0.0996	0.6942 ± 0.0997	< −0.9088 ± 0.0768	0.4491 ± 0.1207

### Features distribution

**Fig 5:**
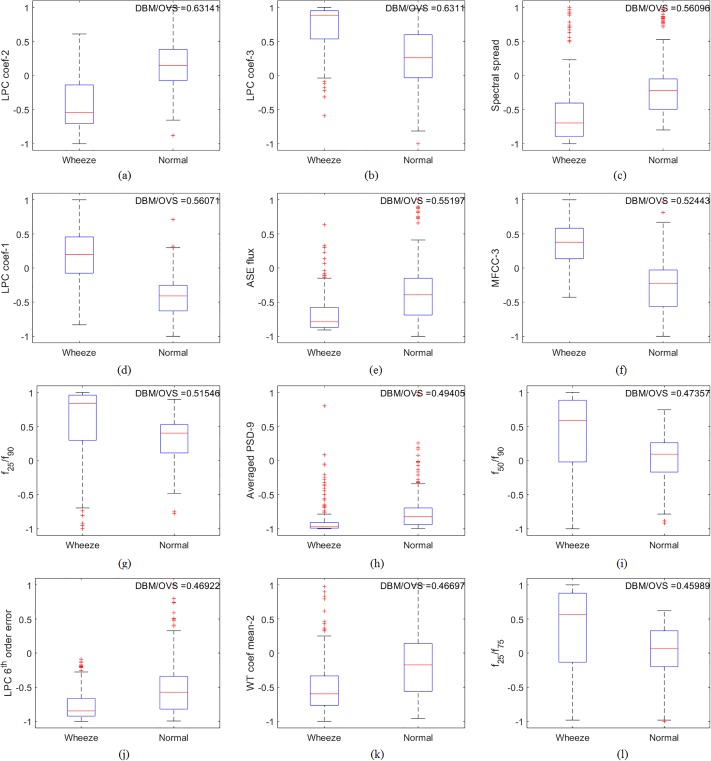
Box Plot of (a) LPC coef-2, (b) LPC coef-3, (c) Spectral spread, (d) LPC coef-1, (e) ASE flux, (f) MFCC-3, (g) *f*_25_/*f*_90_, (h) Averaged PSD-9, (i) *f*_50_/*f*_90_, (j) LPC 6^th^ order error, (k) WT coef mean-2, and (l) *f*_25_/*f*_75_ which represent features with best Distance Between Median (DBM) and Overall Visible Spread (OVS) ratios.

The separation in the feature space between wheezing and normal respiratory sounds was studied in terms of the Distance Between Medians (DBM), as a percentage of Overall Visible Spread (OVS). Fig 5 represents this visually, as box plots of the features with highest DBM/OVS ratios. The corresponding values are listed in Table 6. In this case, LPC coefficient-2 has the highest ratio, followed by LPC coefficient-3 and spectral spread. All box plots shown in Fig 5 have a difference in median values between wheeze and normal breath sounds. However, there are features with overlapping quantiles such as the LPC coef-3 an spectral spread in Fig 5b and 5c respectively. A more ideal feature distribution would have less overlapping quantiles, such as in LPC coef-2 and MFCC-3 in Fig 5a and 5f respectively.

**Table 6:** Features with highest ratio of DBM and OVS.

Feature Name	DBM/OVS Ratio
LPC coef-2	0.6314
LPC coef-3	0.6311
Spectral spread	0.5610
LPC coef-1	0.5607
ASE Flux	0.5520
MFCC-3	0.5244
*f*_25_/*f*_90_	0.5155
Averaged PSD-9	0.4940
*f*_50_/*f*_90_	0.4736
LPC 6th order error	0.4692
WT coef mean-2	0.4670
*f*_25_/*f*_75_	0.4599

### Features ranksum

The Wilcoxon’s ranksum test was used to measure the values distribution of the all the features. This is a nonparametric test of null hypothesis considering that a value randomly selected from one group when compared to a value randomly selected from the other group would have equal probability of being higher or lower. In this study, ranksum test was performed on the features extracted from the first breath event from each recording. The test results can be seen in Table 7, where features with the highest effect size are listed. The effect size of the ranksum test was measured by using the common language effect size definition [36]. Compared to other features, tonality index obtained the highest effect size. The highest effect size was found to be 0.8795. This represents the ratio of all possible pairs of wheeze and normal groups which reject the null hypothesis. The lowest p-value was also achieved by this feature, representing the highest significance compared to other features.

**Table 7:** Features with highest effect size using ranksum test.

Feature Name	Ranksum	p-value	Effect Size
Tonality Index	891	2.9902E-06	0.8795
LPC coef-2	861	3.7751E-05	0.8348
*f*_25_/*f*_90_	963	5.1789E-05	0.8289
LPC coef-1	963	5.1789E-05	0.8289
Spectral irregularity	962	5.6003E-05	0.8274
*f*_50_/*f*_90_	961	6.0541E-05	0.8259
MFCC-3	959	7.0681E-05	0.8229
LPC coef-3	951	1.2964E-04	0.8110
Averaged PSD-16	841	1.7423E-04	0.8051
Averaged PSD-17	835	2.6887E-04	0.7961
Spectral flatness	830	3.8260E-04	0.7887
Averaged PSD-18	824	5.7813E-04	0.7798

### Computation time of features

In certain time-critical or real-time applications, the computation time of features is an important constraint. This is heavily dependent on the processor speed and architecture. However, a relative comparison can still be made by computing all the features on the same processor. When considering different domains, time-domain features can be extracted fastest as they do not require any domain transformation. This is not the case for cepstral, spectral, and wavelet domain features. To transform from time to cepstral domain, a Discrete Cosine Transform (DCT) is required, while for spectral domain, a modified periodogram or some other form of transformation is needed.

To illustrate the differences in computation time between features, the average time needed to extract a feature from data of average length of 829.1 ms was determined. This average was computed based on a simulation run of 100 iterations and the times reported were rounded to the nearest 0.1 *μs*. All the computations were performed on a standard Desktop Computer with an Intel Core i7-4790 CPU 3.6GHz 3.6GHz, with 16.0 GB of RAM, running MATLAB version 2017a. The results for the fastest feature in each domain are shown in Table 8.

**Table 8:** Features with fastest average computation time in each domain.

Domain	Feature	Time (ms)
Time	RMS	0.0102
Spectral	Tonality index	0.3850
Cepstral	MFCC	1.4987
Wavelet	WT coef energy	1.5847

### Optimal combination of features

In certain applications, the accuracy and reliability provided by one feature for detection of wheezes is not enough. These applications necessitate the use of a combination of multiple features to achieve the desired performance. However, the top features may not give the most optimum performance when used together. It is therefore important to determine which features work together in combination to achieve the best performance. To evaluate the combination of features best suited to discriminate between wheeze and normal respiratory sounds, a binary logistic regression model is used. The accuracy of this classifier is ascertained in the test dataset, using both F1 score and MCC metrics, in all possible combinations of two and three features.

Tables 9 and 10 show the best average performance when two features are used for classification. It can be observed from Table 9 that the combination of tonality index and spectral slope achieved the highest F1 score. This is a 4.56% increase in F1 score value compared to when only tonality index was used. Similarly, the addition of the spectral slope feature to the tonality index feature increased the MCC from 0.5790 to 0.7161. This is shown in Table 10.

**Table 9:** F1 score of the best pairs of features for wheeze and normal sound classification using logistic regression on the Test-Set.

First Feature	Second Feature	F1 Score
Tonality index	Spectral slope	0.8319
MFCC-3	MFCC-1	0.8165
MFCC-3	Spectral slope	0.8154
Tonality index	MFCC-1	0.8152
MFCC-3	RMS	0.8133
MFCC-3	Averaged PSD-2	0.8112
MFCC-3	Entropy mean	0.8108
Entropy difference	Spectral Irregularity	0.8099
MFCC-3	Spectral Irregularity	0.8089
MFCC-10	Spectral Irregularity	0.8080
MFCC-3	Entropy ratio	0.8078
MFCC-3	Spectral skewness	0.8049
Tonality index	-	0.7863
MFCC-3	-	0.7786

**Table 10:** MCC of the best pairs of features for wheeze and normal sound classification using logistic regression on the Test-Set.

First Feature	Second Feature	MCC
Tonality index	Spectral slope	0.7161
Tonality index	MFCC-1	0.7154
Tonality index	Entropy ratio	0.7142
Spectral slope	*f*_25_/*f*_90_	0.7053
MFCC-1	MFCC-2	0.6981
Entropy difference	Spectral Irregularity	0.6933
Tonality index	MFCC-8	0.6849
Spectral crest factor	Spectral Irregularity	0.6849
Spectral slope	MFCC-2	0.6823
Tonality index	Averaged PSD-7	0.6822
Tonality index	Renyi entropy	0.6811
Tonality index	MFCC-11	0.6788
Tonality index	-	0.5790
*f*_25_/*f*_90_	-	0.5420

It is also important to take into account the additional time and complexity needed when adding more features. The addition of spectral slope to tonality index in Table 9, while increasing the F1 score by 4.56%, needed 2.025 ms of average processing time which means a 1.64 ms addition to extracting tonality index alone. Combining MFCC-1 and MFCC-3 on the other hand, which achieved the second best F1 score in Table 9, needed only 1.4987 ms on average. Addition of either spectral slope or MFCC-1 to tonality index improved the MCC to 0.7161 or 0.7154 respectively. The additional time for the feature extraction for this improvement was 1.64 ms and 1.4987 ms, representing a 426% and 389% increase respectively.

The addition of a third feature into the feature set can further improve the performance of the LRM classifier. The best performing features, when used in combination of three, are shown in Tables 11 and 12, as optimized using the highest F1 score and MCC respectively. In this case, the highest F1 score was achieved by the combination of MFCC-3, tonality index, and ZCR features at 87.18%. Further, in comparison with the combination of tonality index and spectral slope, which achieved the highest F1 score for two features (Table 9), an improvement of 1.4% was obtained with the addition of MFCC-3. When using MCC to determine the best combination of features, the combination of tonality index, spectral slope, and averaged PSD-17, resulted in the highest performance. Note that the first two features in this case were the same as those in Table 10 (best combination when two features are used), and the addition of averaged PSD-17 improved the MCC significantly from 0.7161 to 0.7267.

**Table 11:** F1 score of the best three features vectors for wheeze and normal sound classification using logistic regression on the Test-Set.

First Feature	Second Feature	Third Feature	F1 Score
MFCC-3	Tonality index	ZCR	0.8718
MFCC-3	Tonality index	RMS	0.8596
MFCC-3	WT coef STD-4	WT coef mean-4	0.8595
MFCC-3	Tonality index	Spectral slope	0.8533
MFCC-3	Tonality index	Entropy ratio	0.8533
MFCC-3	Entropy ratio	Spectral Irregularity	0.8485
Tonality index	Spectral slope	Spectral kurtosis	0.8479
Tonality index	Spectral slope	Spectral flatness	0.8462
MFCC-3	Entropy ratio	Entropy mean	0.8458
Tonality index	STD	Spectral slope	0.8451
Tonality index	Spectral crest factor	Spectral slope	0.8447
MFCC-3	WT coef mean ratio-4	ZCR	0.8438

**Table 12:** MCC of the best three features vectors for wheeze and normal sound classification using logistic regression on the Test-Set.

First Feature	Second Feature	Third Feature	MCC
Tonality index	Spectral slope	Averaged PSD-17	0.7267
Tonality index	Spectral slope	WT coef mean ratio-1	0.7246
Tonality index	MFCC-3	ZCR	0.7201
Tonality index	Spectral slope	Spectral crest factor	0.7199
Tonality index	Spectral slope	MFCC-5	0.7190
Tonality index	Spectral slope	Spectral flatness	0.7169
Tonality index	Spectral slope	Averaged PSD-18	0.7154
Tonality index	Spectral slope	STD	0.7154
MFCC-10	Spectral slope	*f*_25_/*f*_90_	0.7126
Tonality index	MFCC-1	Entropy ratio	0.7120
WT coef energy-5	MFCC-3	Spectral irregularity	0.7120
Tonality index	Spectral slope	Averaged PSD-11	0.7115

**Fig 6:**
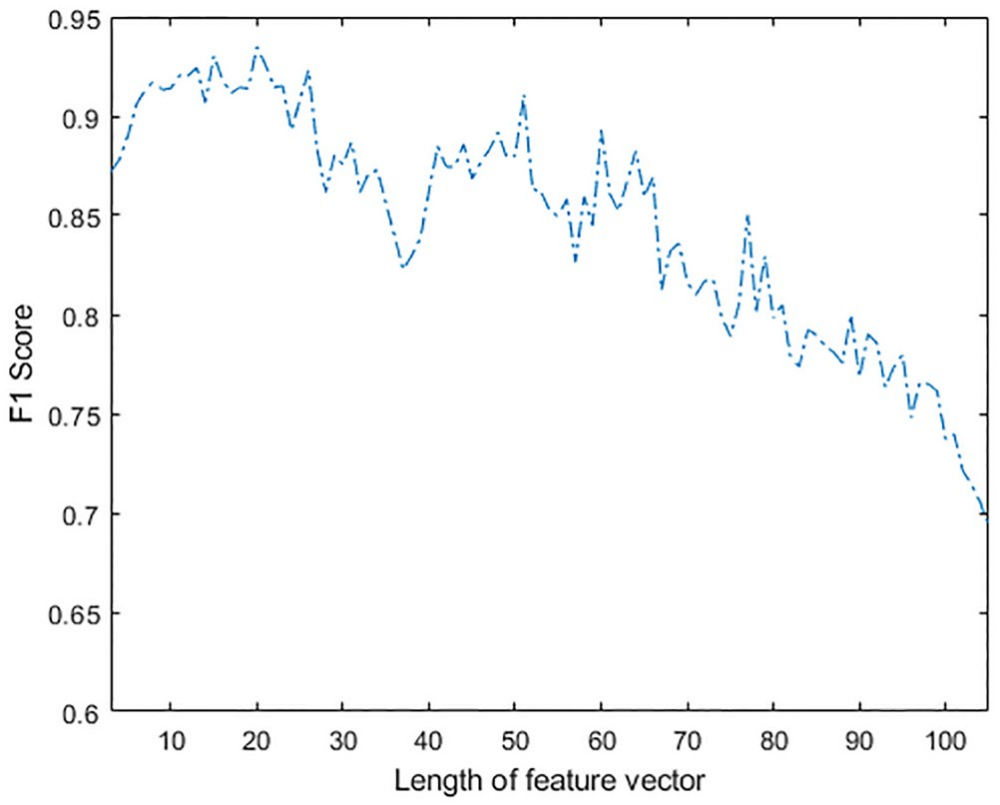
Effect of feature vector length on F1 Score of LRM.

**Fig 7:**
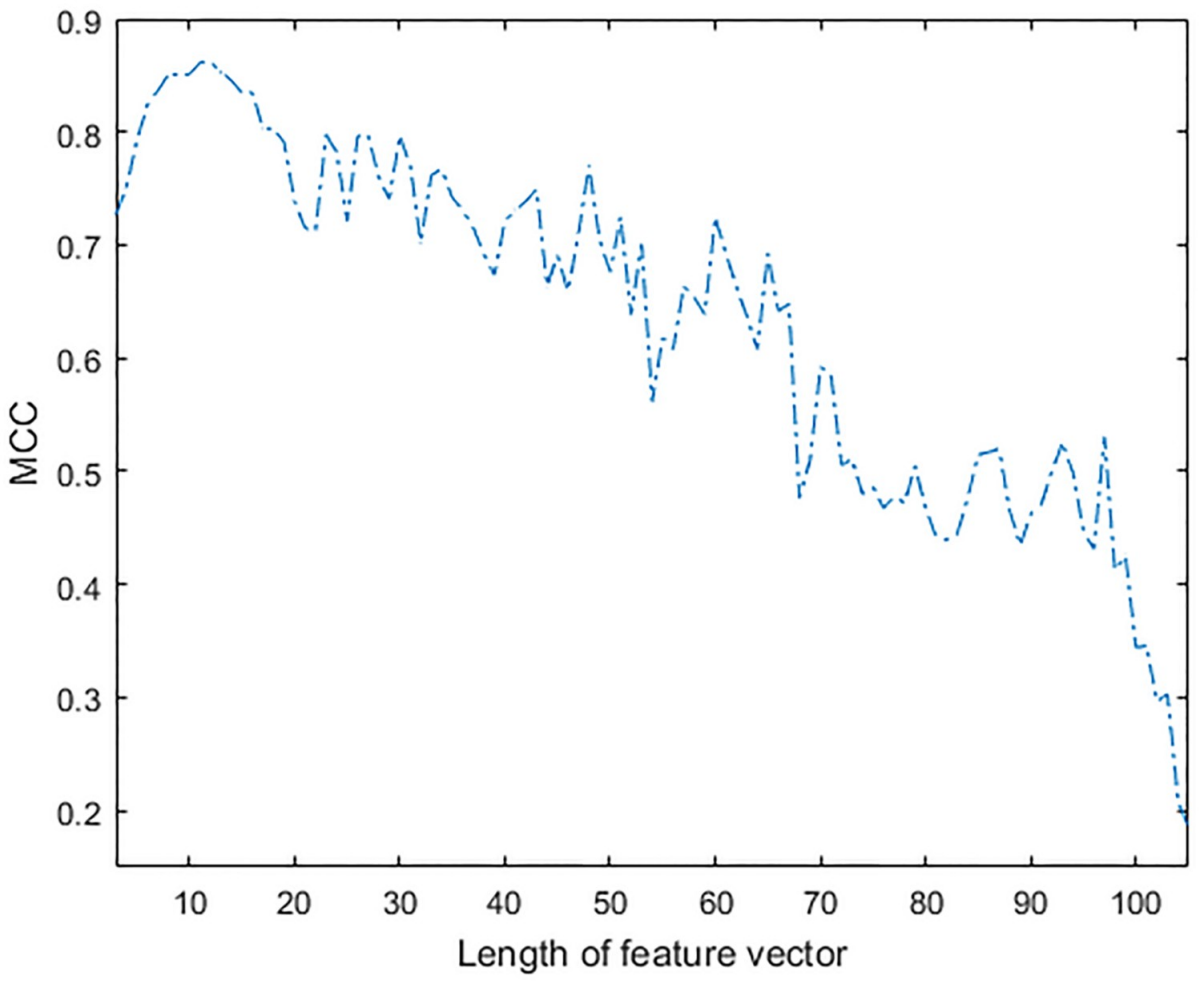
Effect of feature vector length on MCC of LRM.

It is clear that the addition of features helps to improve the classification accuracy, but there comes a point when introducing a new feature has very little impact in performance and may in fact have a detrimental effect on classification accuracy. Hence, to further evaluate the effect of increasing feature vector size on the classification performance, a sequential feature selection method was used to evaluate the F1 score and MCC of the LRM classifier. The effect of additional features on performance, when optimized using F1 score and MCC is shown in Figs 6 and 7 respectively. An overall improvement in F1 score can be observed in Fig 6 when up to twenty features were combined. Improvement of the MCC shown in Fig 7 was observed up to the point when a feature vector of length eleven was used. The addition of other features beyond this point did not improve the performance of the classifier. The performance of the classifier on test dataset was actually reduced when further features were added. This was possibly caused by over-fitting problems in the model, as the new features may not be adding any more information to the test dataset.

## Discussion

This paper evaluated the performance of different features for automated detection of wheezes from respiratory sounds. The top performing features were determined using different objective functions and performance metrics. The rational for doing this was that the *best* feature is usually different in various applications and is heavily dependent on a number of constraints resulting in a number of trade-offs. For example, features with better F1 score would be more useful when the trade-off between sensitivity and number of false positives is important. Similarly, a higher MCC value for a feature generally represents a more balanced overall performance.

The top three features when optimized using the AUC and F1 score were MFCC-3, LPC coef-1, and tonality index. Using MCC as the metric, LPC coef-1 came out on top together with tonality index and MFCC-3 features. The fastest feature to compute was the RMS energy but this resulted in poor classification performance, with F1 Score of 0.7018 and MCC of 0.3373. MFCC-3, LPC coef-1, and tonality index achieved better performance in all three different metrics, AUC, F1 score, and MCC, compared to other features. This could be explained by the randomness of normal respiratory sounds when compared to wheezes. Tonality index measures the unpredictability of a spectrum [21]. Similarly, LPC coef-1 correspond to how well a previous sample in predicting the current sample, while MFCC measures how a cosine function with certain periodicity can be fitted to the log spectrum of the signal. In this study, the computation time comparison was made by measuring the average time for extracting each feature individually. In future work, it may be useful to expand on this and look at the their computational complexity and storage requirements for efficient real-time implementation. Different optimisation methods may also be used such as implementing the algorithm in fixed-point arithmetic which may reduce computational burden in extracting features. However, it is important to note that these would also be dependent on the hardware architecture chosen for any such implementation.

Compared to other features, LPC coef-2 was shown to have the highest DBM/OVS ratio. Ranksum hypothesis test was performed on all features, with tonality index achieving the lowest p-value with highest effect size. This represents the highest significance, as can be seen in Table 7. A total of 87.95% of all possible pairs of features, from wheeze and from normal respiratory sounds support the hypothesis that the features extracted from wheezes have higher value than when extracted from normal respiratory sounds.

Combinations of features were also evaluated using a logistic regression classifier. Logistic regression models were built with all possible pairs of feature combinations. The best performance was obtained when tonality index was paired with spectral slope feature, achieving the highest F1 Score. When three features were combined, the highest F1 Score, 87.18%, was obtained by combining MFCC-3, tonality index, and ZCR. The results and features were different when MCC was used to find the optimal threshold. This reinforces the fact that multiple metrics are needed to characterize the performance of each feature and the metric used must be carefully selected by evaluating the requirements of any given application.

The classifier used to evaluate the features in this study is very simple since the purpose is to demonstrate the discriminatory capabilities of individual features. This is useful for small, battery-powered, low-resource devices designed for long term use. In these cases, it is desirable to reduce the computational requirements by using simpler classifiers with minimum number of features. This also helps to reduce power consumption and extend the system battery life. In applications where computational requirements are not severely limited, the overall classification performance can nevertheless be improved using more advanced classifiers such as support vector machines or artificial neural networks together with a combination of several features.

Automatic detection of wheezing sounds is needed as part of symptom monitoring in management of CRDs, specifically in asthma and COPD. The ability to discriminate between wheezing and normal respiratory sounds using a simple method would be beneficial especially when continuous monitoring using devices with limited power-budget is needed. The use of a single feature and a simple linear threshold classifier would help to reduce the computational burden, thus prolonging the usability of the monitoring device. By reducing complexity, a monitoring device could also be developed that is smaller in size, lighter in weight, and easier to use.

## Conclusion

This paper has presented a comprehensive evaluation of the discriminatory abilities of different types of time, spectral, wavelet, and cepstral features with a total size of 105 for automatic identification of wheezes in breathing. It has been demonstrated that certain individual features (MFCC, tonality index) are much more accurate in detection of wheezes. However their computation requirements are higher than those of simpler time-domain features. In addition, it has also been shown that while the use of multiple features does increase the classification accuracy in some cases, the gain in performance becomes very limited after a certain number of features. While the classifier used in this work is very simple, the use of other more complex classifiers such as support vector machines, artificial neural networks, etc. may help to increase the classification performance at the added cost of computational complexity. Thus, it is important to take all the competing requirements into account when selecting a feature for wheeze detection in different applications. The results presented in this paper will provide highly useful insights to address these requirements for the development of wheeze detection algorithms.
